# Heterogeneity of regional inflection points from pressure-volume curves assessed by electrical impedance tomography

**DOI:** 10.1186/s13054-019-2417-6

**Published:** 2019-04-16

**Authors:** Gaetano Scaramuzzo, Savino Spadaro, Andreas D. Waldmann, Stephan H. Böhm, Riccardo Ragazzi, Elisabetta Marangoni, Valentina Alvisi, Elena Spinelli, Tommaso Mauri, Carlo Alberto Volta

**Affiliations:** 1 Department of Morphology, Surgery and Experimental Medicine, Intensive Care Unit, University of Ferrara, Azienda Ospedaliera - Universitaria Sant’Anna Hospital, Via Aldo Moro, Ferrara, Italy; 2 Department of Anesthesiology and Intensive Care Medicine, Rostock University Medical Center, Rostock, Germany; 3 Department of Anesthesia, Critical Care and Emergency, Fondazione IRCCS (Istituto di Ricovero e Cura a Carattere Scientifico) Ca’ Granda, University of Milan, Milan, Italy

**Keywords:** Pressure-volume curve, Electrical impedance tomography, Mechanical ventilation, Acute respiratory failure, acute respiratory distress syndrome, Personalized medicine

## Abstract

**Background:**

The pressure-volume (*P*-*V*) curve has been suggested as a bedside tool to set mechanical ventilation; however, it reflects a global behavior of the lung without giving information on the regional mechanical properties. Regional *P*-*V* (PVr) curves derived from electrical impedance tomography (EIT) could provide valuable clinical information at bedside, being able to explore the regional mechanics of the lung. In the present study, we hypothesized that regional *P*-*V* curves would provide different information from those obtained from global *P*-*V* curves, both in terms of upper and lower inflection points. Therefore, we constructed pressure-volume curves for each pixel row from non-dependent to dependent lung regions of patients affected by acute hypoxemic respiratory failure (AHRF) and acute respiratory distress syndrome (ARDS).

**Methods:**

We analyzed slow-inflation *P*-*V* maneuvers data from 12 mechanically ventilated patients. During the inflation, the pneumotachograph was used to record flow and airway pressure while the EIT signals were recorded digitally. From each maneuver, global respiratory system *P*-*V* curve (PVg) and PVr curves were obtained, each one corresponding to a pixel row within the EIT image. PVg and PVr curves were fitted using a sigmoidal equation, and the upper (UIP) and lower (LIP) inflection points for each curve were mathematically identified; LIP and UIP from PVg were respectively called LIPg and UIPg. From each measurement, the highest regional LIP (LIPr_MAX_) and the lowest regional UIP (UIPr_MIN_) were identified and the pressure difference between those two points was defined as linear driving pressure (Δ*P*_LIN_).

**Results:**

A significant difference (*p* < 0.001) was found between LIPr_MAX_ (15.8 [9.2–21.1] cmH_2_O) and LIPg (2.9 [2.2–8.9] cmH_2_O); in all measurements, the LIPr_MAX_ was higher than the corresponding LIPg. We found a significant difference (*p* < 0.005) between UIPr_MIN_ (30.1 [23.5–37.6] cmH_2_O) and UIPg (40.5 [34.2–45] cmH_2_O), the UIPr_MIN_ always being lower than the corresponding UIPg. Median Δ*P*_LIN_ was 12.6 [7.4–20.8] cmH_2_O and in 56% of cases was < 14 cmH_2_O.

**Conclusions:**

Regional inflection points derived by EIT show high variability reflecting lung heterogeneity. Regional *P*-*V* curves obtained by EIT could convey more sensitive information than global lung mechanics on the pressures within which all lung regions express linear compliance.

**Trial registration:**

Clinicaltrials.gov, NCT02907840. Registered on 20 September 2016.

**Electronic supplementary material:**

The online version of this article (10.1186/s13054-019-2417-6) contains supplementary material, which is available to authorized users.

## Background

Although mechanical ventilation (MV) might be necessary to maintain adequate gas exchanges in patients with acute respiratory failure, ventilator-induced lung injury (VILI) can amplify local and systemic inflammation and contribute to disease progression [1–3]. VILI is caused by multiple mechanisms such as high volumes, high pressures, and cyclic opening and closing of the peripheral airways [4]. Moreover, lungs with patchy infiltrates are heterogeneous and have considerable parenchyma loss of volume available for ventilation [5]. The gravitational increasing weight of the parenchyma and the inhomogeneous inflammation process create differences between lung units located in different regions of the lungs, with different distending pressures, dynamic behavior, and therefore physiologic needs [6, 7]. Information coming from mechanical respiratory properties assessed through airway pressure (Paw) characterize the global behavior of the lung but possibly cannot identify regional peculiarities.

The pressure-volume (*P*-*V*) curve is a respiratory monitoring technique that explores changes in the respiratory system compliance along a wide range of Paw (e.g., between 0 and 40 cmH_2_O). A *P*-*V* curve usually has sigmoidal shape [8] with two inflection points—the lower (LIP) and the upper (UIP)—and an almost linear part in between. The physiological interpretation of the *P*-*V* curve classically considers the region between the boundaries of the LIP and UIP as safe for mechanical ventilation (i.e., a PEEP level higher than LIP and plateau pressure lower than UIP) to prevent both atelectrauma and barotrauma [9, 10]. However, one can question whether that this approach is able to prevent VILI since it considers the respiratory system as a whole and does not take into account the local mechanical behavior which is influenced by gravity and super-imposed pressures [11]. In fact, it is possible that the same airway pressure might overinflate parts of the lung while being harmless for others or that a certain level of positive end-expiratory pressure (PEEP) is able to keep parts of the lung ventilated not being enough for others.

Electrical impedance tomography (EIT) is a radiation-free lung imaging technique, which allows continuous bedside monitoring of the regional mechanical properties by measuring changes in impedance associated with ventilation [12–15]. In the present study, we hypothesized that regional *P*-*V* curves would provide different information from those obtained from global *P*-*V* curves, both in terms of upper and lower inflection points. To confirm this hypothesis, we constructed pressure-volume curves for each pixel row from non-dependent to dependent lung regions of patients affected by acute hypoxemic respiratory failure (AHRF) and acute respiratory distress syndrome (ARDS) [16].

## Methods

### Patient enrollment

We performed a new and additional analysis of data collected during a prospective study carried out in the intensive care unit (ICU) of Azienda Ospedaliera Universitaria Sant’Anna (Ferrara, Italy) between December 2015 and October 2016. We enrolled adult patients (aged ≥ 18 years) with AHRF or ARDS, who were deeply sedated and paralyzed as per clinical decision with a ratio of partial pressure of oxygen in arterial blood to fraction of inspired oxygen (PaO_2_/FiO_2_) ≤ 300 mmHg and clinical PEEP ≥ 5 cmH_2_O. Exclusion criteria were (a) refusal to participate to the study, (b) pregnancy, (c) pulmonary cardiogenic edema, (d) unstable hemodynamics (defined by a systolic arterial pressure of 90 mmHg or less or mean arterial pressure of 60 mmHg or less), (e) pneumothorax, (f) severe chronic obstructive pulmonary disease, (g) impossibility to correctly position the EIT belt (e.g., chest drainage, surgical wound dressings), and (h) contraindications to EIT monitoring (e.g., pacemaker, automatic implantable cardioverter defibrillator). The ethical committee of Ferrara, (protocol no. 141285) Italy, approved the study, and informed consent was obtained following local regulations. At enrollment, we collected demographic and clinical data of each patient.

### Patient monitoring

Patients were in supine position, deeply sedated with propofol and morphine, and paralyzed with rocuronium bromide during the study protocol. A heated pneumotachograph (Fleisch no. 2, Fleisch, Lausanne, Switzerland) was positioned at the airway opening to record Paw and flow. Volumes were derived by integrating the flow signal. An oblique textile electrode belt with 32 active electrodes was placed around the chest along the 4th and the 6th intercostal space, and EIT recordings were performed using the Swisstom BB^2^ system (Swisstom, Landquart, Switzerland) [17]. The EIT device selected the dimension of the belt based on the circumference of the patient’s hemithorax and adapted the thorax contour according to the entered height, weight, and gender. Heart rate, arterial pressure, SpO_2_, and EtCO_2_ were continuously monitored during the study. Additional details have been published in a previous study [18].

### Pressure-volume maneuvers

Each *P*-*V* curve maneuver was carried out using the constant slow-flow inflation technique, as previously described [19]. During the inflation, the pneumotachograph was used to record flow and Paw while the EIT signals were digitally recorded on a dedicated USB drive. The *P*-*V* curves were performed at three different PEEP levels: 5 cmH_2_O, 10 cmH_2_O, and 15 cmH_2_O. At each measurement, we monitored also hemodynamic and respiratory parameters (peak pressure, plateau pressure, PEEPtot, respiratory system compliance), obtained by an end-inspiratory and end-expiratory occlusions [18].

### Regional pressure-volume curves and data analysis

The new analysis was performed only on measurements simultaneously recorded by EIT and pneumotachograph. For each pressure-volume curve maneuver, a global *P*-*V* curve (PVg) was built, plotting Paw versus volume. EIT dynamic images recorded during inflation were analyzed as follows: to obtain regional *P*-*V* curves, the impedance change (Δ*Z*) in the predefined lung region of the dynamic EIT images was grouped into regions of interest (ROIs), according to the gravitational vector, in a craniocaudal direction (region 1: non-dependent area, region 1 + *n*: dependent lung). The analysis was conducted in 19 gravity-dependent regions of interest, each one corresponding to a row of pixels. On each ROI, the Δ*Z* during the slow inflation maneuver was plotted against the corresponding Paw. In this way, we obtained regional *P*-*V* (PVr) curves, one for each ROI [19–22]. To compare different ROIs, both Δ*V* and Δ*Z* during the maneuver were normalized to 1. Lower and upper inflection points were estimated by fitting a sigmoidal equation (Eq. 1) into the PVg, as previously described [8].

1 $$ V=a+b+{e}^{-\left(P-c\right)/d} $$

To fit the PVr, we adapted Eq. 1 assuming that Δ*Z* = Δ*V* (Eq. 2); we also limited the values of *a* and *b* to keep their value in a physiological range.

2 $$ \Delta  Z=a+b+{e}^{-\left(P-c\right)/d} $$

The pressures at which the function rapidly changes slope can be defined from the intersections between a tangent to the *P*-*V* curve at the point of maximal compliance (*P* = *c*) and the lower (lower inflection point, LIP) and upper (upper inflection point, UIP) asymptotes, respectively. In Eqs. 1 and 2, these points can be obtained as:

$$ {\displaystyle \begin{array}{c}\mathrm{LIP}=c-2d\\ {}\mathrm{UIP}=c+2d\end{array}} $$

From each measurement, we therefore obtained the following data:

- One fitted equation for the PVg and *n* equations for the PVr (*n =* number of ROIs). From each equation, we obtained global (global UIP = UIPg; global LIP = LIPg) and regional inflection points (regional UIP = UIPr; regional LIP = LIPr)
- The difference in each ROI between the UIPr and LIPr, defined as regional range of linear compliance (*R*_LC_)
- The minimum UIPr, corresponding to the lowest level of pressure linked to the disappearance of the linear part of the corresponding PVr, defined as UIPr_MIN_
- The maximum LIPr, corresponding to the highest level of pressure linked to the start of the linear part of the corresponding PVr, defined as LIPr_MAX_
- The average of UIPr for each measurement (UIPr_AVE_)
- The average of LIPr for each measurement (LIPr_AVE_)
- The pressure difference between the LIPr_MAX_ and the UIPr_MIN_ was defined as linear driving pressure (Δ*P*_LIN_); this value corresponds to a pressure interval in which every PVr is on its linear part

**Fig. 1:**
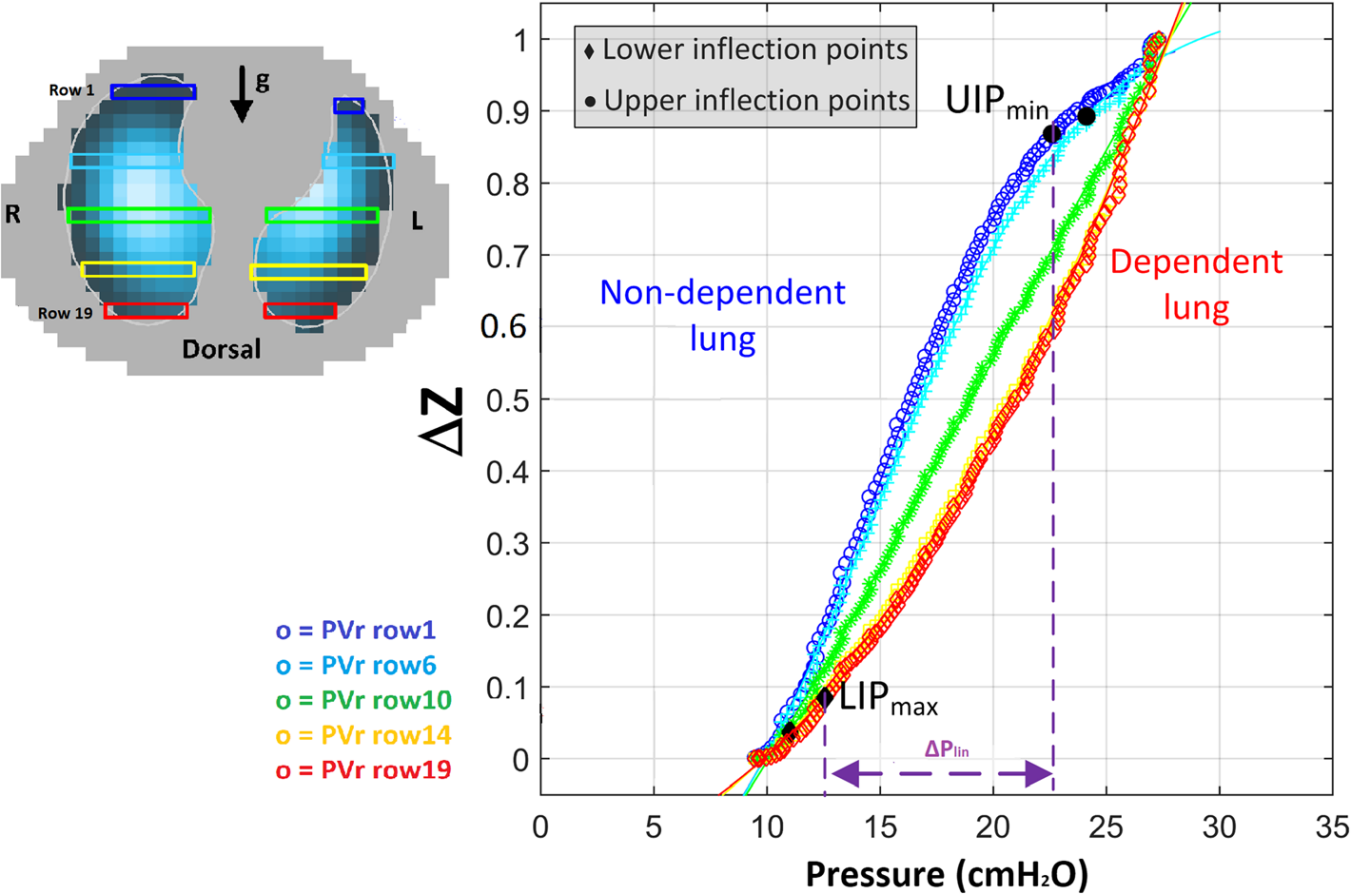
Example of regional pressure-volume curves. A slow inflation maneuver was recorded simultaneously using electrical impedance tomography and a pneumotachograph. Representative patient (patient # 2, PEEP10 cmH_2_O)

The regional *P*-*V* curves were considered valid if the quality of the fitting *R*^2^ > 0.9. Measurements with negative inflection points were considered invalid. An illustrative analysis of one measurement from a representative patient is shown in Fig. 1.

### Statistics

Data are presented as median and interquartile range (IQR). Wilcoxon matched-pairs signed rank test was used to evaluate differences between paired data. Mann-Whitney test was used to compare unpaired groups of data. Statistical analyses were performed using SPSS 20.0 statistical software (IBM, Armonk, NY, USA) and GraphPad Prism 6 for Windows (GraphPad Software, La Jolla, CA, USA, www.graphpad.com). In all statistical analyses, a two-tailed test was performed and the *p* value equal or less than 0.05 was considered statistically significant.

## Results

### Patients’ characteristics and analysis

**Fig. 2:**
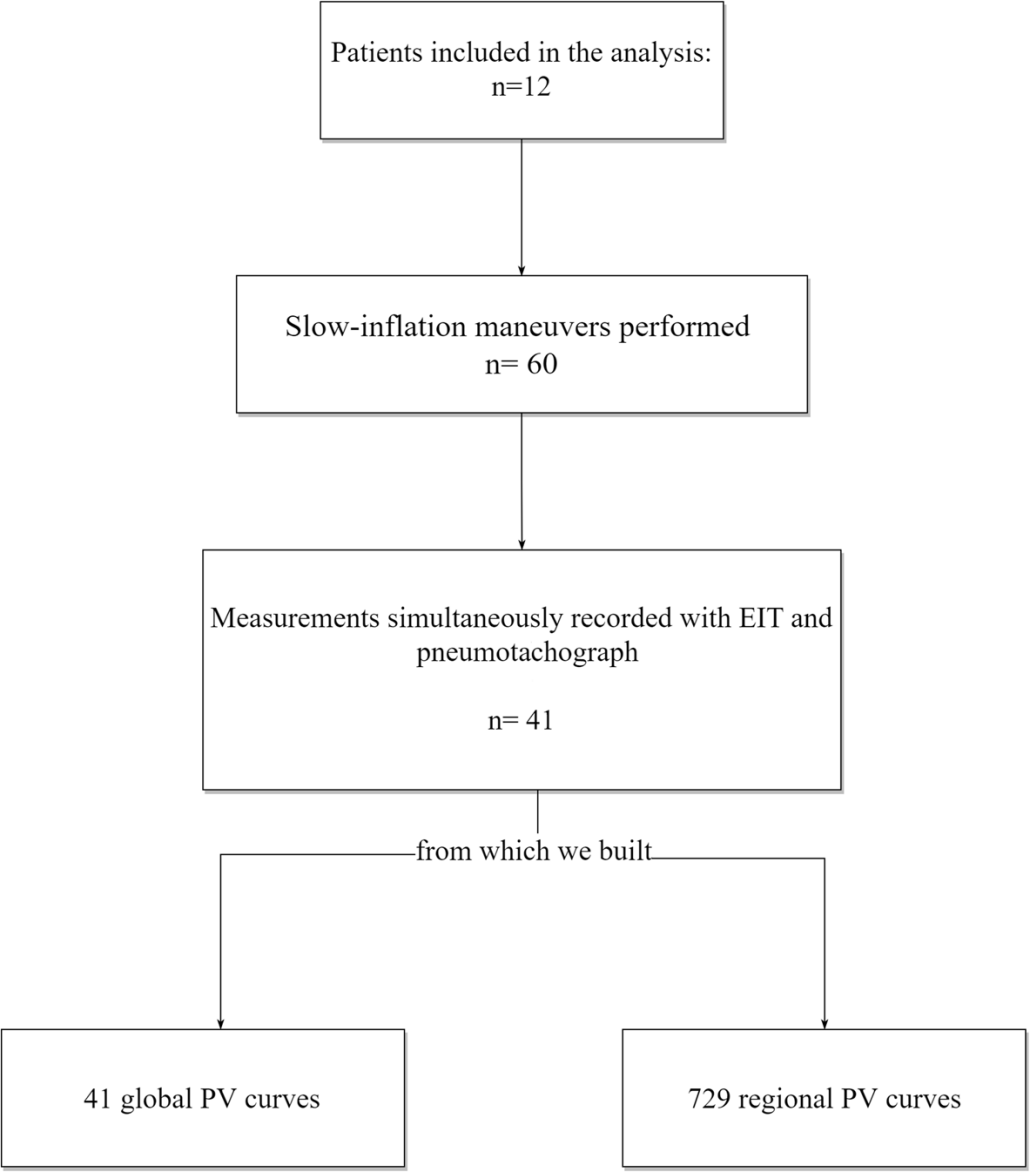
Analysis flow chart

Patients’ characteristics are summarized in Table 1. Of note, 5/12 (42%) had ARDS. Forty-one measurements from 12 patients were considered of sufficient technical quality for the analysis. The PaO_2_/FiO_2_ at enrollment was 218 [170–262] cmH_2_O, and the median clinical PEEP used was 7.5 cmH_2_O. From the 41 measurements, we built 729 PVr and 41 PVg (Fig. 2).

**Table 1:** Patients’ main characteristics

Patient	Gender	Age (years)	BMI	SAPS II (at ICU admission)	SOFA (day of study)	Etiology of acute respiratory failure	Days of intubation	ARDS (yes or no)	PaO_2_/FiO_2_(mmHg)*	PEEP (cmH_2_O)*	Outcome
1	F	75	25	34	7	Sepsis	7	Yes	198	10	Survivor
2	M	79	26	33	8	Thoracic trauma	7	Yes	160	8	Non-survivor
3	M	90	29	46	7	Sepsis	1	Yes	205	10	Survivor
4	M	71	29	30	10	Postoperative respiratory failure	2	No	230	7	Survivor
5	F	80	35	22	9	Postoperative respiratory failure pneumonia	5	Yes	263	8	Survivor
6	M	69	33	30	4	Postoperative respiratory failure	2	No	168	8	Survivor
7	M	66	24	40	6	Sepsis	1	No	294	6	Survivor
8	F	85	19	38	5	Septic shock	1	No	273	7	Survivor
9	F	80	33	63	10	Hemorrhagic shock	4	Yes	256	10	Survivor
10	F	76	24	33	8	Hemorrhagic shock	2	No	258	7	Non-survivor
11	F	72	26	38	10	Postoperative respiratory failure pneumonia	6	No	175	6	Survivor
12	F	78	35	38	11	Postoperative respiratory failure	4	No	141	6	Survivor
Median [IQR]	5 M/7 F	77 [71–80]	28 [24–33]	36 [31–40]	8 ± [6.3–10]		3 [1.3–5.8]	5 yes/7 no	218 [170–262]	7.5 [6.3–9.5]	2 non-survivors/10 survivors

*BMI* body mass index, *SAPSII* simplified acute physiology score II, *ICU* intensive care unit, *SOFA* sequential organ failure assessment, *ARDS* acute respiratory distress syndrome, *PaO*_*2*_*/FiO*_*2*_ partial pressure of arterial oxygen on inspired fraction of oxygen ratio, *PEEP* positive end-expiratory pressure

*Before starting the protocol (clinical)

### Regional and global lower inflection points

**Fig. 3:**
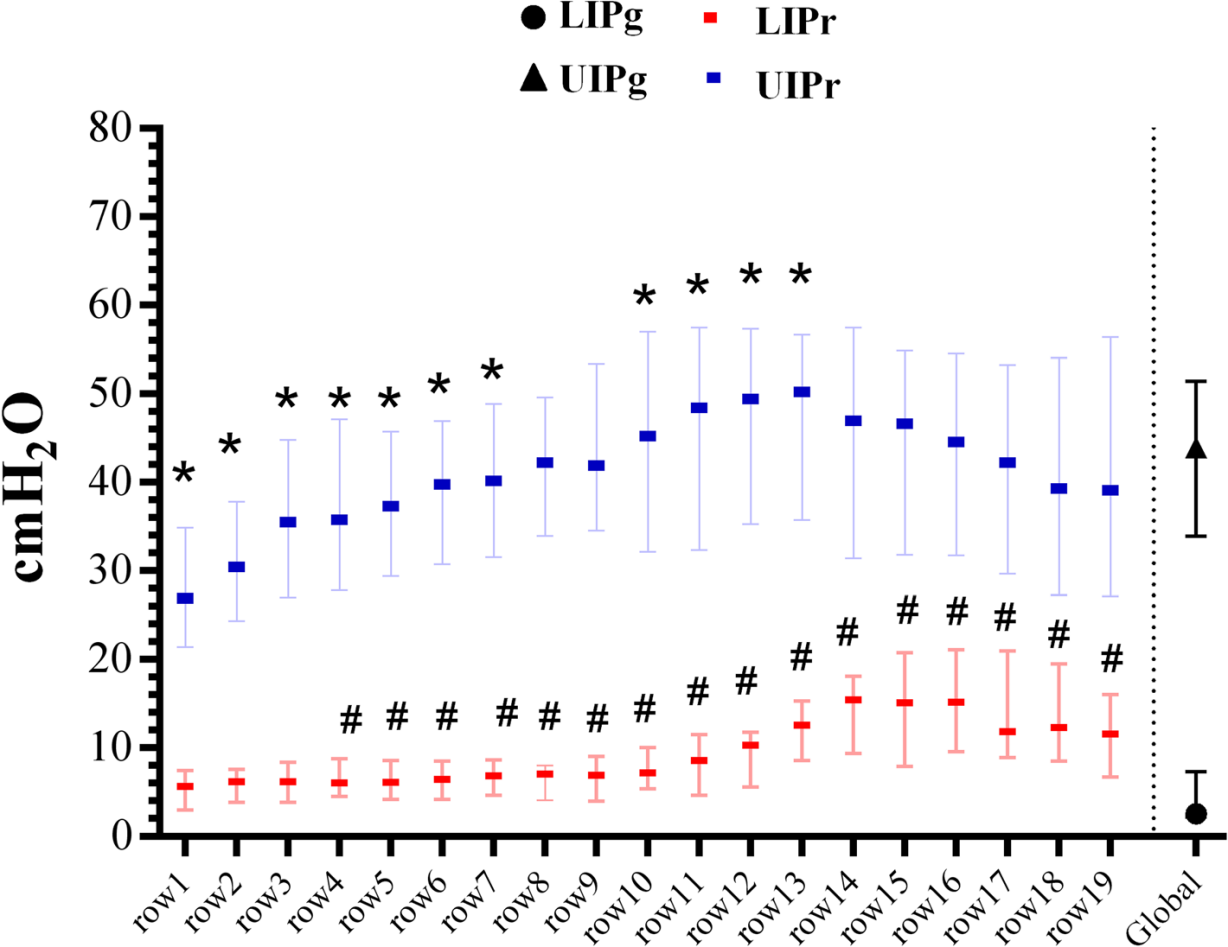
Results: regional and global inflection points. Regional inflection points are in gravitational order (row 1 = most non-dependent; row 19 = most dependent). Values are expressed as median and interquartile range. Asterisk denotes different from UIPg (*p* < 0.05); number sign denotes different from LIPg (*p* < 0.05)

**Fig. 4:**
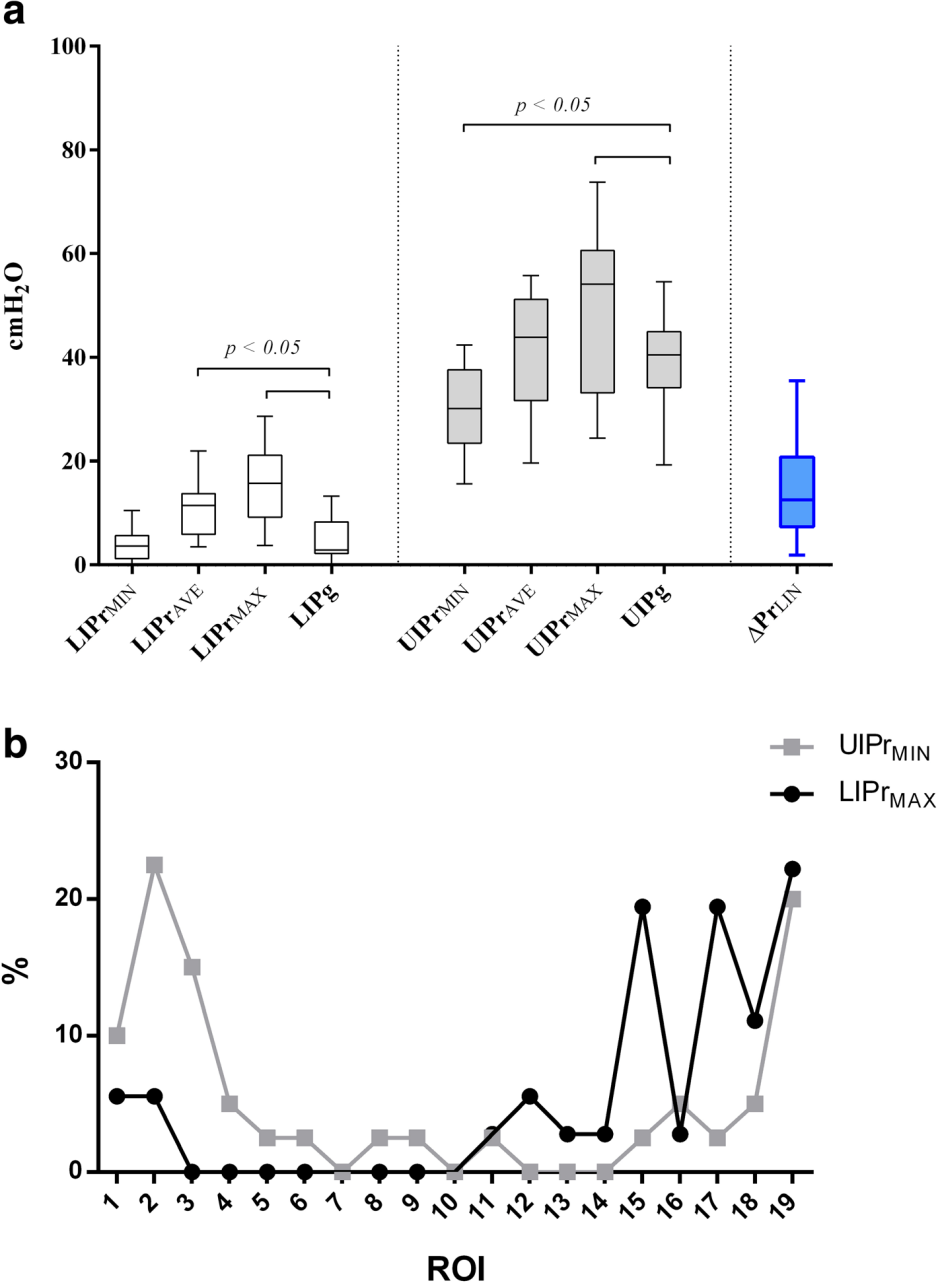
Results: comparison between regionally derived parameters, global inflection points, and location of LIPr_MAX_ and UIPr_MIN_. **a** Values expressed as median [IQR] of LIPr_MAX_, LIPr_MIN_, LIPr_AVE_, LIPg, UIPr_MAX_, UIPr_MIN_, UIPr_AVE_, UIPg, and ∆Pr_LIN_. LIPr_MAX_ and UIPr_MIN_ were respectively different from LIPg and UIPg (Mann-Whitney unpaired *t* test). **b** Cumulative distribution of UIPr_MIN_ and LIPr_MAX_ position; ROI 1, non-dependent; ROI 1 + *n*, dependent lung. In 55% of the measurements, the UIPr_MIN_ was positioned within the first five ROIs (non-dependent lung); in 75% of the measurements, the LIPr_MAX_ was positioned within the last five ROIs (dependent lung)

A valid LIPg was found in 37% of measurements; its median value was 2.9 [2.2–8.9] cmH_2_O. LIPr values increased from the non-dependent to the dependent lung (Fig. 3). Comparing LIPr to the LIPg, we found statistically significant difference in rows 4–19 but not in the least dependent ones (rows 1–3). A statistically significant difference was also found between the LIPg and LIPr_MAX_ (15.8 [9.2–21.1] cmH_2_O; *p* < 0.05). LIPr_MAX_ was in the last five most dependent ROIs in 77% of measurements (Fig. 4).

### Regional and global upper inflection points

The median UIPg value was 40.5 [34.2–45] cmH_2_O. A statistically significant difference was found between UIPg and UIPr_MIN_ (30.1 [23.5–37.6] cmH_2_O; *p* < 0.05), with UIPr_MIN_ located in the four most non-dependent regions in 55% of measurements (Fig. 4). A supplemental analysis, based on the level of PEEP, can be found in the supplemental digital content (Additional file 1: Table S1).

### Regional linear driving pressure

The pressure interval between LIPr_MAX_ and UIPr_MIN_ (ΔPr_LIN_; Fig. 1) was 12.6 [7.4–20.8] cmH_2_O (Fig. 3). In 56% of measurements, ΔPr_LIN_ was lower than 14 cmH_2_O. The pressure interval between each regional LIPr and UIPr (*R*_LC_) is reported in Additional file 2: Figure S1; we found a high variability in this value, which was higher in the central lung and lower in both the most dependent and the non-dependent lung.

## Discussion

The main results of this study are as follows: (1) The global *P*-*V* and the regional *P*-*V* curves do not provide the same information, and inflection points of the global *P*-*V* curve are different from those of the PVr (i.e., higher LIP and lower UIP); (2) the analysis of the regional *P*-*V* curves allows to detect the heterogeneity of the inflection points of the different lung regions; regional inflection points are gravity dependent with UIPr_MIN_ located in the most non-dependent lung and LIPr_MAX_ in the most dependent; (3) the “safe” limit of pressure between LIPr_MAX_ and UIPr_MIN_ was reduced compared to the one obtained by global *P*-*V* curve (Table 2).

**Table 2:** Results

Number of measurements	41
LIPg	2.9 [2.2–8.9]
LIPr_MIN_	3.7 [1.2–5.7]
LIPr_MAX_	15.8 [9.25–21.1]*
LIPr_AVE_	11.5 [5.9–13.8]*
UIPg	40.5 [34.2–45]
UIPr_MIN_	30.1 [23.5–37.6]^#^
UIPr_MAX_	54.2 [33.2–60.6]^#^
UIPr_AVE_	43.9 [31.7–51]
ΔPr_LIN_	12.6 [7.4–20.8]

Data expressed as median [IQR]. Mann-Whitney test (unpaired *t* test)

*Different from LIPg (*p* < 0.01)

^#^Different from UIPg (*p*≥ 0.01)

Different attempts have been made in the last 30 years for use of the *P*-*V* curve of the respiratory system to set mechanical ventilation [9, 10]. The inflection points of this curve express changes in compliance, reflecting a rise in ventilated alveolar units—due to alveolar recruitment or airway opening or both, or a pressure that exceed intrinsic PEEP—(for LIP) and the overstretching of already ventilated lung (for UIP). Hence, setting mechanical ventilation in the linear part of the curve between these two points could be protective against both intra-tidal alveolar opening and closing (atelectrauma) and overinflation (barotrauma/volutrauma). Despite the physiological background, this approach considers the lung as a homogenous mono-compartimental system without taking the entity of regional inflammation [23] and the gravitational superimposed pressure on each region into account [11].

On the one hand, the LIP of the global *P*-*V* curve has been classically considered the critical opening pressure at which alveoli start to open or to be fully recruited but this concept has recently been questioned. A mathematical model of ARDS [24] and CT scans on patients affected by ALI/ARDS [25] showed that recruitment of new units occurs also beyond the LIP, influencing the slope of the *P*-*V* curve. Moreover, alveolar opening pressure (i.e., recruitment) is not a fixed threshold but a continuous range of pressure, progressively increasing from the non-dependent to the dependent lung. Finally, LIP could represent reversal of airway closure rather than alveolar recruitment [26]. We confirmed these findings since we found a gradient of regional lower inflection points from the non-dependent to the dependent lung: moving toward the vertebral-dependent lung, the LIPr increases, probably as an effect of the highest alveolar (or small airways pressure) pressure needed to overcome the resultant superimposed pressure (Fig. 3). Moreover, we found a significant difference between the LIPg, the regional minimum LIP (LIPr_MIN_), and the LIPr in the most non-dependent lung (ROIs 1, 2, 3). Therefore, the LIP on the global *P*-*V* curve may express the behavior of the uppermost compartment that receives air at the beginning of inflation, as already suggested by Hickling using a mathematical model [24]. This would, however, imply that information about the LIPr in the central and dependent lung is not adequately reflected by the global *P*-*V* curve. Since the dependent lung is the region most affected by intra-tidal recruitment and airway closure due to the higher pressure needed to start inflation, using the global LIP—that expresses the behavior of the non-dependent lung—to set PEEP would leave large dependent regions prone to atelectrauma. Our data show that the minimal pressure needed to ventilate above all regional LIP (LIPr_MAX_) was 15.8 [9.25–21.1] cmH_2_O, a value much higher than that derived from the LIPg. If we hypothesize that the ideal ventilation should occur on the linear part of each regional *P*-*V* curve and that the starting point of the respiratory cycle is PEEP, then LIPg would underestimate the value to achieve this target.

On the other hand, alveolar overdistension leads to compression of pulmonary vessels and capillaries, disruption of alveolar epithelium, and physical breaks in endothelial plasma membranes [27] triggering the proinflammatory signaling cascade which results in inflammation, edema, and cell death [28]. Titrate tidal volume to keep a plateau pressure below the “overdistension threshold” can be useful to avoid lung inflammation.

Amato et al. [10] found a massive reduction of 28-day mortality (38% vs 71%) in patients ventilated with low tidal volumes (6 ml/kg/PBW); this finding was later confirmed by large-scale RCTs [29] and meta-analysis [30] proposing low tidal volume ventilation as the standard of care. Despite this potential advantage, PBW is not an accurate index of the actual lung size since it does not express the dimension of the baby lung [5, 31]. The upper inflection point is considered the beginning of lung overstretch and, ideally, could be used to titrate indirectly TV on the lung size. Roupie et al. [9] found that, in a ventilation based on a TV = 10 ml/kg, in 80% of the studied population the TV needed to be reduced to 7.8 ± 0.9 ml/kg to obtain a Pplat below the UIP. We found that UIPg was not able to highlight the regional overstretch since UIPg differed from UIPr_MIN_, a value that corresponds to the lowest pressure at which regional overinflation starts. Moreover, UIPg was different from UIPr in the non-dependent lung (Fig. 3); using the UIPg to limit pressure can therefore underestimate the potential damage on this region.

In our population, UIPr_MIN_ was 30.1 [23.5–37.6] cmH_2_O, not far from the suggested as protective Pplat threshold found in AHRF patients affected by sepsis [32, 33]. In addition, a recent multilevel mediation analysis on 3562 ARDS patients showed that a reduction of Pplat < 30 H_2_O could not further improve survival rate. It is important to underline that in our population the range was wide, from a minimum of 13.9 cmH_2_O to a maximum of 49.7 cmH_2_O, being below 30 cmH_2_O in 44% of measurements. A fixed value, 30 cmH_2_O in this case, cannot represent therefore the universal safe number, suggesting that a tailored safe threshold should be set based on the patient’s characteristics.

In this analysis, we introduced two variables: LIPr_MAX_—the minimal pressure able to overcome all regional lower inflection points—and UIPr_MIN_—the maximal pressure to avoid a ventilation above all regional upper inflection points. We hypothesize that LIPr_MAX_ can be helpful in setting PEEP while UIPr_MIN_ can represent a pressure limit to avoid regional overstretch.

Recently, respiratory driving pressure (DP) has been found to predict mortality in ARDS patients [34] and a titration of TV on DP has been suggested to reduce VILI [35, 36]. The connection between DP and overdistension has been furthermore confirmed by a CT scan study on humans [37]. In the current analysis, we introduced a new variable: Δ*P*_LIN_. Its value reflects the maximal pressure range which avoids regional overinflation and derecruitment, thereby allowing a ventilation simultaneously above every regional LIP and below every regional UIP. Δ*P*_LIN_ expresses, therefore, individual threshold pressures, above which VILI might occur. In our study population, although mean Δ*P*_LIN_ was 12.6 [7.4–20.8] cmH_2_O and therefore close to the value defined as protective in a recent prospective study [38], in 56% of the cases, it was below 14 cmH_2_O, the limit value of driving pressure suggested by Amato et al. [34]. This indicates that, in some patients, a driving pressure threshold of 14 cmH_2_O, despite generally considered protective, would be slightly too high in order to prevent regional damage (Fig. 4).

Our results underline that the traditional *P*-*V* curve could give partial information on the way patients should be ventilated. The linear part of pressure-volume relationship does not pertain to all lung regions, being instead a compromise between the mechanical characteristics of the dependent and not dependent part of the lung. Since it is fundamental to select the correct amount of PEEP to limit atelectrauma, while avoiding overcoming the UIP to limit overdistension, patients could be ventilated in the linear portion of the *P*-*V* curves taking into account all lung regions. We were able to find this linearity in the entire lung regions by simply using the maximal LIP and the minimal UIP. LIP and UIP exhibited a huge variability among patients, underlining that mechanical ventilation must be personalized. It is tempting to say that the analysis of the regional *P*-*V* curves could help in achieving this goal. In particular, it remains to be elucidated whether the VT associated with linear regional DP is able to control CO_2_ and if this new approach could lead to a novel indication for extracorporeal lung assist [39].

Our study has several limitations. Firstly, we performed only inspiratory pressure-volume curve, as it is the most used for evaluating the variability of the compliance of the respiratory system. Alternatively, a physician could examine the expiratory limb of the curve to evaluate closure pressure. However, we decided to use the same methodology used in a RCT to set PEEP using the *P*-*V* curve [10]. Secondly, the study population consisted of a limited number of patients with heterogeneous diseases; therefore, no inferences on the patient outcome can be derived from our data. Thirdly, we did not measure transpulmonary pressure: this tool would have been useful to distinguish between the respiratory system and lung regional inflection points. Fourthly, the lack of randomization of PEEP levels and the return to zero pressure at the end of each step could have influenced the results of this study [18].

## Conclusions

Regional inflection points derived by EIT show high variability, reflecting the heterogeneity of the lung. Regional *P*-*V* curves obtained by EIT could convey more sensitive information than global lung mechanics on pressures within which all lung regions express linear compliance. Future studies will explore if setting mechanical ventilation based on regional *P*-*V* curves could impact on patients’ outcome.

## Additional files

Additional file 1: Regional inflection points from pressure-volume curves assessed by electrical impedance tomography as a guide to mechanical ventilation. Supplemental digital content. Additional results; **Table S1.** LIPg, UIPg, LIPr_MAX_, UIPr_MIN_, and ΔP_LIN_ at the 3 different levels of PEEP. (DOCX 16 kb)

Additional file 2: **Figure S1.** Ventrodorsal distribution of the regional difference between LIPr and UIPr. Regional differences between LIPr and UIPr in the different regions of interests (ROI); ROI1 = non-dependent; ROI1 + n = dependent lung. Values expressed as median [IQR]. (JPG 257 kb)

## Abbreviations

∆*V*: Variation of volume
∆*Z*: Variation of impedance
AHRF: Acute hypoxic respiratory failure
ARDS: Acute respiratory distress syndrome
CT: Computed tomography
DP: Driving pressure
EIT: Electrical impedance tomography
EtCO_2_: End tidal CO_2_
FiO_2_: Inspiratory fraction of oxygen
ICU: Intensive care unit
IQR: Interquartile range
LIP: Lower inflection point
LIPr_MAX_: Regional maximal lower inflection point
MV: Mechanical ventilation
PaO_2_: Arterial partial pressure of oxygen
Paw: Airway opening pressure
PBW: Predicted body weight
PEEP: Positive end-expiratory pressure
PEEPtot: Total PEEP
Pplat: Plateau pressure
*P*-*V*: Pressure-volume
PVg: Global pressure-volume
PVr: Regional pressure-volume
RCT: Randomized controlled trial
*R*_LC_: Regional range of linear compliance
ROI: Region of interest
SpO_2_: Peripheral oxygen saturation
UIP: Upper inflection point
UIPr_MIN_: Regional minimum upper inflection point
USB: Universal serial bus
VILI: Ventilator-induced lung injury
VT: Tidal volume
Δ*P*_LIN_: Regional linear driving pressure
