# Supplementary material for: Heterogeneity of regional inflection points from pressure-volume curves assessed by electrical impedance tomography
Source: Crit Care. 2019 Apr 16;23:119. doi: 10.1186/s13054-019-2417-6 (PMC6469223; doi:10.1186/s13054-019-2417-6)
Supplement: Supplementary file 1 — Regional inflection points from pressure-volume curves assessed by electrical impedance tomography as a guide to mechanical ventilation. Supplemental digital content. Additional results; Table S1. LIPg, UIPg, LIPrMAX, UIPrMIN, and ΔPLIN at the 3 different levels of PEEP. (DOCX 16 kb) [file 13054_2019_2417_MOESM1_ESM.docx]

**Heterogeneity of regional inflection points from pressure volume curves assessed by Electrical Impedance Tomography.**

**Supplemental Digital Content**

**Table S1: LIPg, UIPg, LIPr_MAX_, UIPr_MIN_ and ΔP_LIN_ at the 3 different levels of PEEP.**

|  | **LIPg** | **LIPr_MAX_** | **UIPg** | **UIPr_MIN_** | **ΔP_LIN_** |
| --- | --- | --- | --- | --- | --- |
| **PEEP5 (cmH_2_O)** | 5.6 [2.9.-8.3] | 16.9 [12.2-22.5] | 35.7 [24.8-54.9] | 25.4[20-31.6] | 8.9[7.4-9.2] |
| **PEEP10 (cmH_2_O)** | 2.6 [1.2-10.4] | 16 [10-21.3] | 39.1 [34.2-44.3] | 30.1 [22.6-37.5] | 13.2 [6.4-19.4] |
| **PEEP15 (cmH_2_O)** | 2.9[0.8.-5.6] | 13 [4-16.4] | 42.7 [40.5-45.5] | 34.9 [27.3-45.1] | 18.6 [11.6-33.8] |

Values of Global lower inflection point (LIPg), global upper inflection point (UIPg), regional highest lower inflection point (LIPr_MAX_), regional lowest upper inflection point (UIPr_MIN_) and pressure difference of regional linear compliance (ΔP_LIN_) at the 3 different levels of PEEP. Values expressed as median [IQR].
