# Supplementary figures and images for: Heterogeneity of regional inflection points from pressure-volume curves assessed by electrical impedance tomography
Source: Crit Care. 2019 Apr 16;23:119. doi: 10.1186/s13054-019-2417-6 (PMC6469223; doi:10.1186/s13054-019-2417-6)

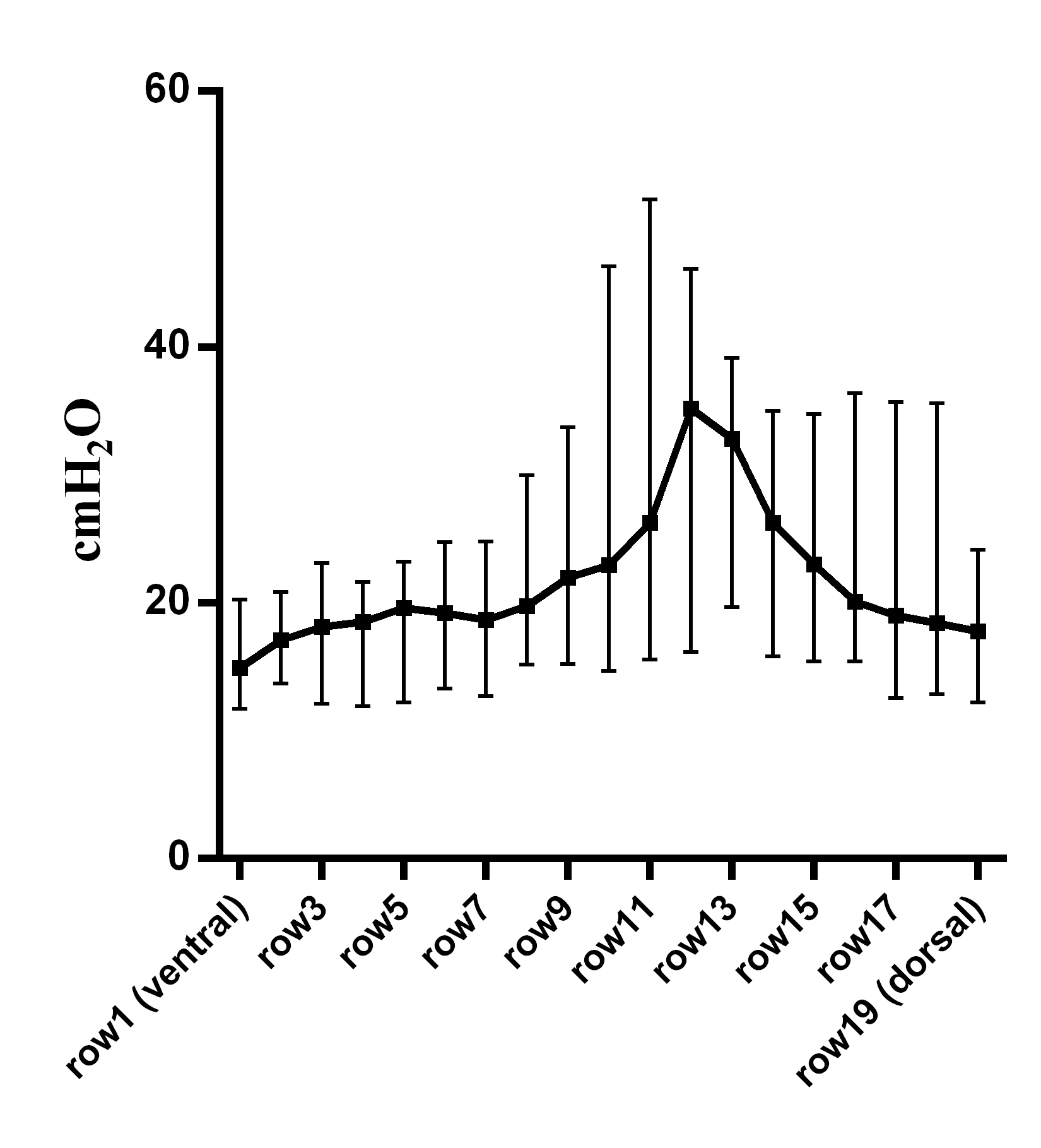

Supplement: Supplementary file 2 — Figure S1. Ventrodorsal distribution of the regional difference between LIPr and UIPr. Regional differences between LIPr and UIPr in the different regions of interests (ROI); ROI1 = non-dependent; ROI1 + n = dependent lung. Values expressed as median [IQR]. (JPG 257 kb) [file 13054_2019_2417_MOESM2_ESM.jpg]
